# Author Correction: Advancing COVID-19 diagnosis with privacy-preserving collaboration in artificial intelligence

**DOI:** 10.1038/s42256-022-00485-5

**Published:** 2022-04-08

**Authors:** Xiang Bai, Hanchen Wang, Liya Ma, Yongchao Xu, Jiefeng Gan, Ziwei Fan, Fan Yang, Ke Ma, Jiehua Yang, Song Bai, Chang Shu, Xinyu Zou, Renhao Huang, Changzheng Zhang, Xiaowu Liu, Dandan Tu, Chuou Xu, Wenqing Zhang, Xi Wang, Anguo Chen, Yu Zeng, Dehua Yang, Ming-Wei Wang, Nagaraj Holalkere, Neil J. Halin, Ihab R. Kamel, Jia Wu, Xuehua Peng, Xiang Wang, Jianbo Shao, Pattanasak Mongkolwat, Jianjun Zhang, Weiyang Liu, Michael Roberts, Zhongzhao Teng, Lucian Beer, Lorena E. Sanchez, Evis Sala, Daniel L. Rubin, Adrian Weller, Joan Lasenby, Chuansheng Zheng, Jianming Wang, Zhen Li, Carola Schönlieb, Tian Xia

**Affiliations:** 1 Department of Radiology, Tongji Hospital and Medical College, Huazhong University of Science and Technology, Wuhan, China; 2 School of Artificial Intelligence and Automation, Huazhong University of Science and Technology, Wuhan, China; 3 Department of Engineering, University of Cambridge, Cambridge, UK; 4 Department of Radiology, Union Hospital of Tongji Medical College, Huazhong University of Science and Technology, Wuhan, China; 5 HUST-HW Joint Innovation Lab, Wuhan, China; 6 CalmCar Inc, Suzhou, China; 7 Wuhan Blood Centre, Wuhan, China; 8 MSA Capital, Beijing, China; 9 The National Centre for Drug Screening, Shanghai Institute of Materia Medica, Chinese Academy of Sciences, Shanghai, China; 10 CardioVascular and Interventional Radiology, Radiology for Quality and Operations, The Cardiovascular Centre at Tufts Medical Centre, Radiology, Tufts University School of Medicine, Medford, OR USA; 11 Russell H Morgan Department of Radiology & Radiologic Science, Johns Hopkins Hospital & Medicine Institute, Baltimore, MD USA; 12 Department of Radiation Oncology, School of Medicine, Stanford University, Palo Alto, CA USA; 13 Department of Radiology, Wuhan Central Hospital, Wuhan, China; 14 Department of Radiology, Wuhan Children’s Hospital, Wuhan, China; 15 Faculty of Information and Communication Technology, Mahidol University, Salaya, Thailand; 16 Thoracic/Head and Neck Medical Oncology, University of Texas MD Anderson Cancer Centre, Houston, TX USA; 17 Translational Molecular Pathology, University of Texas MD Anderson Cancer Centre, Houston, TX USA; 18 Department of Applied Mathematics and Theoretical Physics, University of Cambridge, Cambridge, UK; 19 Oncology R&D at AstraZeneca, Cambridge, UK; 20 Department of Radiology, University of Cambridge, Cambridge, UK; 21 Department of Biomedical Data Science, Radiology and Medicine, Stanford University, Palo Alto, USA; 22 Alan Turing Institute, London, UK; 23 Department of Hepatobiliary Pancreatic Surgery, Affiliated Tianyou Hospital, Wuhan University of Science and Technology, Wuhan, China

**Keywords:** Information systems and information technology, Health care, Computational science, SARS-CoV-2

Correction to: *Nature Machine Intelligence* 10.1038/s42256-021-00421-z, published online 15 December 2021.

In the version of this article initially published, the first name of Chuansheng Zheng was misspelled as Chuangsheng. The error has been corrected in the HTML and PDF versions of the article.

