# Supplementary material for: Advancing COVID-19 diagnosis with privacy-preserving collaboration in artificial intelligence
Source: Nat Mach Intell. Author manuscript; Available in PMC 2024 Jan 23. (PMC10805468; doi:10.1038/s42256-021-00421-z)
Supplement: supplement [file NIHMS1917397-supplement-supplement.pdf]

nature portfolio

https://doi.org/10.1038/s42256-021-00421-z

Supplementary information

Advancing COVID-19 diagnosis with privacy-preserving collaboration in artificial intelligence

In the format provided by the authors and unedited

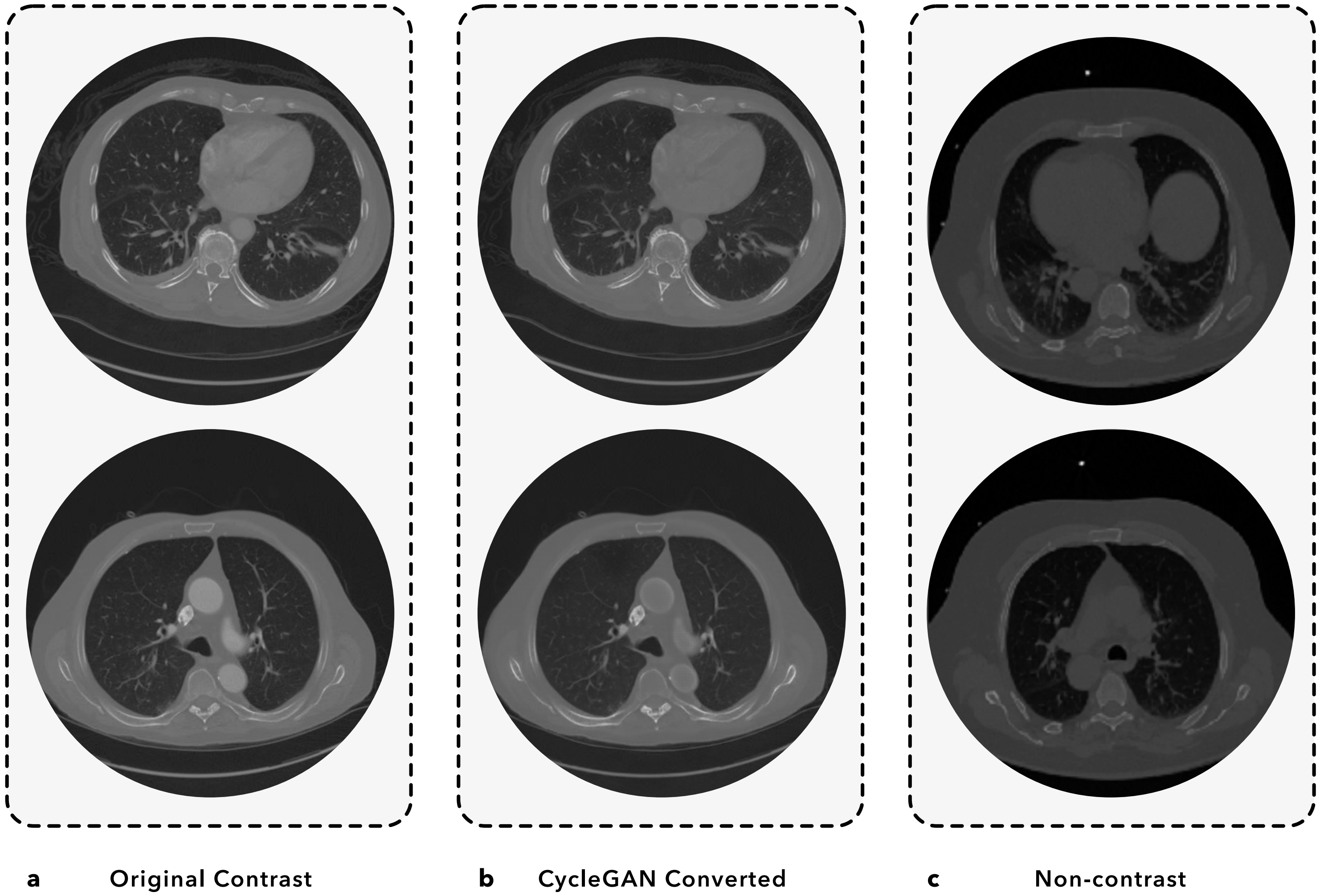

Supplementary Fig. 1 | Conversion of the contrast CTs with CycleGAN. a, Contrast CTs (angiography), **b,** Correspondent converted CTs using CycleGAN to reduce the contrastive regions (pointed out by red arrows), **c,** Non-contrast CTs.

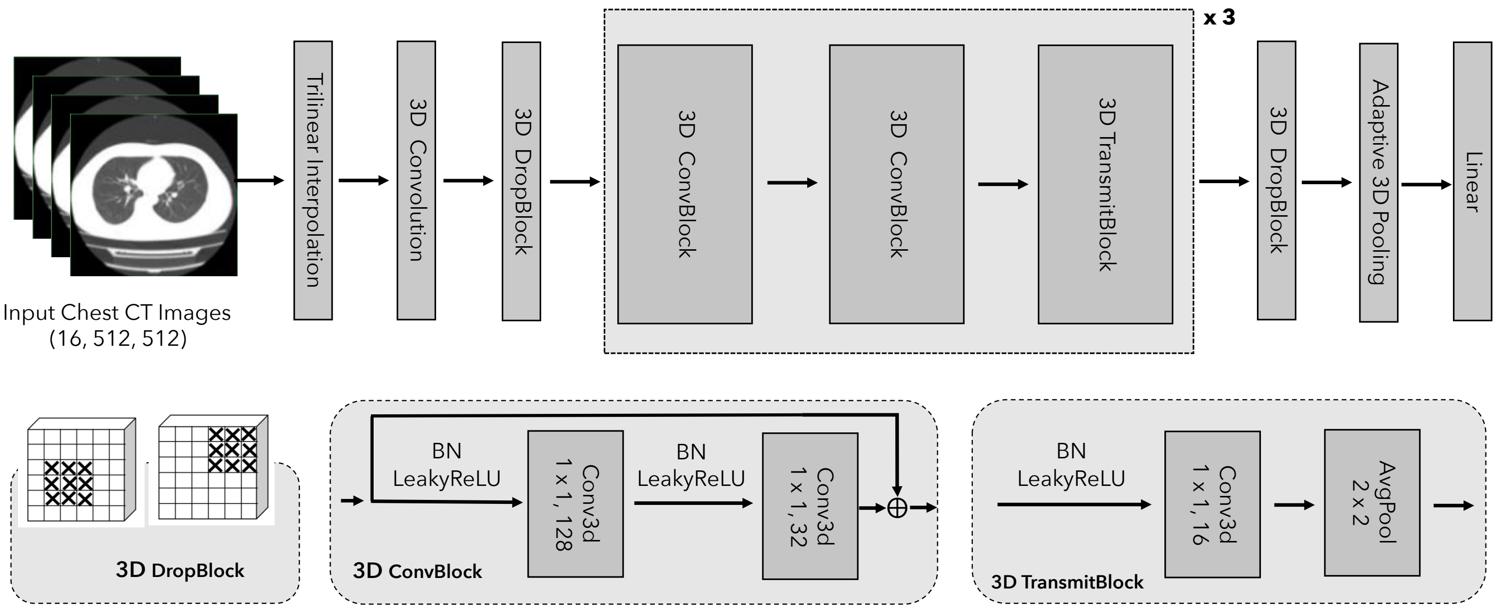

Supplementary Fig. 2 | Architecture of 3D DenseNet.

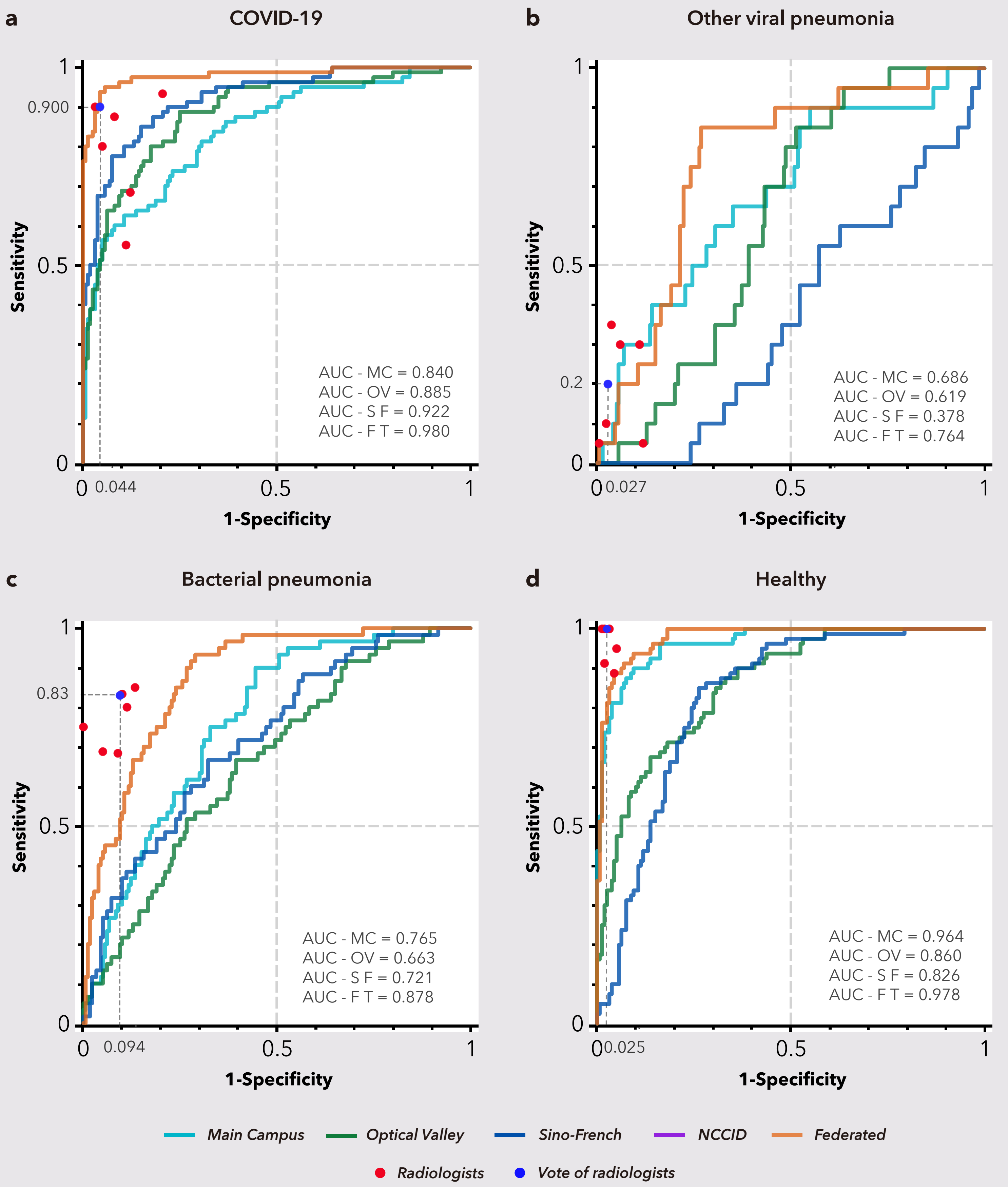

Supplementary Fig. 3 | Detailed model performance on the four-class (a-d) prediction from the China data.

Train split									
Resource	Pneumonia cohort	Gender		Age					Patient amount	CT amount
		Female	Male	0-20	20-40	40-60	60-80	80+		
Main Campus	N/A(healthy)	115	109	18	144	59	3	0	224	727
	COVID-19	66	69	5	39	45	43	3	135	922
	Other viral	26	30	0	8	29	19	0	56	250
	Bacterial	118	136	1	47	104	94	8	254	934
Optical Valley	N/A(healthy)	32	43	8	57	10	0	0	75	278
	COVID-19	54	58	1	16	30	57	8	112	425
	Other viral	0	0	0	0	0	0	0	0	0
	Bacterial	1	12	0	1	3	7	2	13	47
Sino-French	N/A(healthy)	22	21	1	31	10	1	0	43	131
	COVID-19	85	73	2	30	53	66	7	158	853
	Other viral	0	0	0	0	0	0	0	0	0
	Bacterial	10	15	0	8	12	5	0	25	97
								total	**1095**	**4664**

Test split										
Resource	Pneumonia cohort	Gender		Age					Patient amount	CT amount
		Female	Male	0-20	20-40	40-60	60-80	80+		
Main Campus	N/A(healthy)	30	28	3	36	19	0	0	58	191
	COVID-19	21	13	1	5	15	11	2	34	191
	Other viral	8	11	0	4	9	6	0	19	72
	Bacterial	23	27	0	9	20	21	0	50	170
Optical Valley	N/A(healthy)	6	6	0	9	3	0	0	12	44
	COVID-19	13	10	0	6	5	11	1	23	88
	Other viral	0	0	0	0	0	0	0	0	0
	Bacterial	1	1	0	0	1	1	0	2	8
Sino-French	N/A(healthy)	5	5	0	9	1	0	0	10	27
	COVID-19	15	22	0	5	12	19	1	37	244
	Other viral	1	0	0	0	1	0	0	1	12
	Bacterial	2	6	1	1	3	3	0	8	29
Tianyou	COVID-19	-	-	-	-	-	-	-	645	645
Union	COVID-19	-	-	-	-	-	-	-	506	506
								total	**1405**	**2227**

Supplementary Table 1 | Demographical and clinical information of the CN data.

Training and validation split										
**CT Info**	**Pneumonia cohort**	**Gender**		**Age**					**Patient amount**	**CT amount**
		Female	Male	0-20	20-40	40-60	60-80	80+		
Contrast	N/A(healthy)	46	67	0	1	11	62	43	276	1097
	COVID-19	56	88	0	4	35	68	37	145	491
Non-Contrast	N/A(healthy)	21	24	0	1	5	29	10	116	394
	COVID-19	21	31	0	0	7	28	17	54	163
								total	**591**	**2145**

Test split										
**Resource**	**Pneumonia cohort**	**Gender**		**Age**					**Patient amount**	**CT amount**
		Female	Male	0-20	20-40	40-60	60-80	80+		
Contrast	N/A(healthy)	29	42	0	0	4	40	27	160	259
	COVID-19	32	51	0	2	20	39	22	83	138
Non-Contrast	N/A(healthy)	11	18	0	0	4	14	9	75	103
	COVID-19	11	16	0	1	4	19	5	27	37
								total	**345**	**537**

Hospitals Include: Royal United Hospitals Bath NHS Foundation Trust, Brighton and Sussex University Hospitals NHS Trust, London North West University Healthcare NHS Trust, George Eliot Hospital NHS Trust, Cwm Taf Morgannwg University Health Board, Hampshire Hospitals NHS Foundation Trust, Betsi Cadwaladr University Health Board, Ashford and St Peter's Hospitals, Royal Cornwall Hospitals NHS Trust, Sheffield Children's NHS Foundation Trust, Liverpool Heart and Chest Hospital NHS Foundation Trust, Norfolk and Norwich University Hospitals NHS Foundation Trust, Royal Surrey NHS Foundation Trust, Sandwell and West Birmingham NHS Trust, West Suffolk NHS Foundation Trust, Somerset NHS Foundation Trust, Cambridge University Hospitals NHS Foundation Trust, Imperial College Healthcare NHS Trust

Supplementary Table 2 | Demographical and clinical information of the UK data. Part of the cohort demographics is not recorded by some NCCID partnership hospitals.

Test Split		Training and Validation Split			
		Non-contrast		Contrast	
		Real	Real + Synthetic	Real	Real + Synthetic
Non-contrast	Sensitivity	0.703	0.784	0.324	
	Specificity	0.961	0.961	0.864	
	AUC	0.882	0.921	0.655	
Contrast	Sensitivity	0.269		0.914	0.810
	Specificity	0.897		0.916	0.933
	AUC	0.667		0.979	0.931

Supplementary Table 3 | COVID-19 pneumonia identification performance of CNN models trained on contrast and non-contrast split of NCCID dataset (UK). "Real + Synthetic" means the training and validation images include the original split (non-contrast/contrast) as well as the synthesized ones from their couterpart (contrast/non-contrast) via CycleGAN. We conduct no modification on the test set.

Method	CouncilGAN	ACL-GAN	CycleGAN (ours)
Performance (Sensitivity / Specificity)	0.458 / 0.692	0.524 / 0.279	0.703 / 0.784

Supplementary Table 4 | Comparison on the unpaired image translation methods.

Radiologist Expert (Name Abbr.)		ZL	LYM	YZL	COX	HLM	GC	Majority Vote
Healthy	sensitivity	1	1	0.91	0.89	0.95	1	1
	specificity	0.99	0.98	0.98	0.96	0.95	0.97	0.98
COVID-19	sensitivity	0.88	0.9	0.8	0.55	0.68	0.93	0.9
	specificity	0.92	0.97	0.95	0.89	0.88	0.79	0.96
Other viral pneumonia	sensitivity	0.1	0.35	0.3	0.05	0.3	0.05	0.2
	specificity	0.97	0.96	0.94	0.88	0.89	1.0	0.97
Bacterial pneumonia	sensitivity	0.83	0.68	0.85	0.8	0.69	0.75	0.83
	specificity	0.9	0.91	0.87	0.89	0.95	1	0.91
COVID vs non-COVID	sensitivity	0.88	0.90	0.55	0.80	0.68	0.93	0.90
	specificity	0.92	0.97	0.89	0.95	0.88	0.79	0.96

Supplementary Table 5 | Detailed diagnoses from radiologist.

Model	3D-ResNet50	3D-ResNet101	3D-Xception	3D-DenseNet (ours)
Performance (Sensitivity / Specificity)	0.888 / 0.500	0.894 / 0.512	0.831 / 0.525	**0.926** / **0.538**
Model size (# Params)	46.16 M	85.21 M	22.43 M	**0.73 M**

Supplementary Table 6 | Comparison on the 3D CNN models.

Model	3D-ResNet50	3D-ResNet101	3D-Xception	3D-DenseNet (ours)
FLOPs	20.32G	30.31G	35.19G	143.50G

Supplementary Table 7 | Computation cost of 3D CNN models.

Hold-out Data	Cohorts (Positive/Negative)	Performance (Sensitivity)		
		Main Campus	NCCID	FL
Wuhan Union Hospital	506 / 0	0.44	0.53	0.66
Wuhan Tianyou Hospital	645 / 0	0.42	0	0.99

Supplementary Table 8 | Model generalisation on the hold-out dataset.

	Taxonomy (Pneumonia type)	Main Campus	Optical Valley	Sino-French	NCCID	Federated
Four class	healthy - 0	[18, 11, 2, 49]	[27, 39, 0, 14]	[15, 22, 0, 43]	[68, 12, 0, 0]	[76, 1, 3, 0]
	covid - 1	[0, 45, 0, 35]	[1, 77, 0, 2]	[2, 71, 0, 7]	[29, 51, 0, 0]	[1, 78, 1, 0]
	other viral - 2	[0, 1, 0, 19]	[1, 11, 0, 8]	[1, 5, 0, 14]	[13, 7, 0, 0]	[0, 4, 5, 11]
	bacterial - 3	[0, 20, 0, 58]	[5, 40, 0, 15]	[6, 14, 0, 40]	[41, 19, 0, 0]	[14, 5, 12, 29]
Two class	non-covid - 0	[146, 14]	[70, 90]	[119, 41]	[122, 38]	[150, 10]
	covid - 1	[35, 45]	[3, 77]	[9, 71]	[29, 51]	[2, 78]

Supplementary Table 9 | Confusion matrices of locally/federatively trained models.
